# The evaluation of progress in the treatment of traumatic brain injury 

**Published:** 2015

It is well known by specialists and others that traumatic brain injury (TBI) is the main cause of death in young people under 40 years old and, at the same time, represents the cause of approximately two-thirds of post-traumatic deaths.

Data from the World Health Organization show that 11,5 million Europeans who have survived traumatic brain injuries currently present physical disabilities or cognitive and emotional disorders.

The main types of traumatic brain injuries include the following: brain fractures, brain contusions, brain dilacerations, diffuse axonal injuries, and extradural, subdural and intracerebral hematomas. The official data show a growing incidence of the traumatic brain injuries.

Annually, there are almost 245,000 individuals hospitalized with such traumas, and 66,000 of these patients die due to complications.

The number of cases of traumatic brain injury in Romania is continually rising. With an incidence of 300 cases per 100,000 citizens, our country annually registers over 60,000 new TBI cases. Every 8 minutes, a Romanian has a traumatic brain injury. TBI represents 36-40% of the total traumas, and when the association with other traumas is considered, the share rises to 60-65%. Moreover, TBI represents 25-30% of the invalid segment of the population. In addition, 75% of the individuals who die due to road accidents have a traumatic brain injury.

According to the official data, Romania is at the top of the list for TBI among the EU countries, with a death rate due to road accidents of 92 deaths per one million citizens, which is greater than the average of the number of 52 deaths for other countries. 

Just as **Prof. Dafin Mureșanu, MD,** the President of the Romanian Society of Neurology (RSN) and President of the Society for the Study of Neuroprotection and Neuroplasticity (SSNN) declared ***“The number of traumatic brain injuries is continually rising due to many factors, starting with the development of the auto industry and the way technology evolves in transportation and ending with the growth of the rate of domestic accidents. From the data we have at present, we can state that the incidence oscillates between 1,4 million and 1,6 million in Europe, with most of the victims being treated in emergency situations. Traumatic brain injuries must be treated seriously and carefully because they usually have critical medical-social, economic and judicial implications. However, Romania faces some problems in these cases. First, there is a lack of neuropsychologists. We do not have specialists in memory recovery. It is not enough to be able to move again. It is at least as important to be able to communicate and reintegrate in the society. There are only a few centers in Romania in which the patient is treated by multidisciplinary neurorehabilitation teams. There should be a center in every town and a community neurorehabilitation strategy, and a private system of alternative care should be developed.”***

TBI treatment has undergone exponential development the field of neurological recovery, which has obviously generated an acceleration of research efforts during the last several years.

The new discoveries in TBI and also in cognitive recovery, together with many other important themes, represent the main subject of one of the most important medical events of the year 2014. The event is the ***12th Annual Conference of the Academy for Multidisciplinary Neurotraumatology (AMN), organized by the Society for the Study of Neuroprotection and Neuroplasticity (SSNN),*** which took place in Dubai, during the 14th and 17th of November 2014.

***“The event had a large number of participants, including medical personalities from Europe, Asia and the United States of America, and had a complex scientific program, which included actual themes regarding the evaluation of the progress achieved in the treatment of traumatic brain injuries. The importance of the Congress was great because it reunited people with a vast professional experience who have managed to integrate the information offered from interdisciplinary perspectives as a whole”,*** the distinguished **Prof. Dafin Mureșanu, MD,** declared.

The Society for the Study of Neuroprotection and Neuroplasticity (SSNN) was founded in 2005 by an international team of doctors and researchers at the initiative of Prof. Dafin Mureșanu, MD, from “Iuliu Hațieganu” University of Medicine and Pharmacy in Cluj-Napoca and Prof. Ovidiu Băjenaru, MD, from “Carol Davila” University of Medicine and Pharmacy in Bucharest. It is a scientific organization dedicated to fundamental and clinical research with the purpose of creating a discussion forum for a better understanding of the endogen neurobiological processes, as well as the development of pharmacological and non-pharmaceutical therapeutic strategies in the field of neuroprotection and neuroregeneration.

The Congress was very important because it took place only two weeks from another resonant event: The **10th Edition of the Congress of the Society for the Study of Neuroprotection and Neuroplasticity,** which was held in Athens and was organized by the Society for the Study of Neuroprotection and Neuroplasticity (SSNN) and presented in the Editorial of the last issue of the Journal of Medicine and Life, 2014.

Romania was represented in the 12th AMN Congress 2014 by Prof. Dafin Fior Mureșanu, MD, the President of the Society for the Study of Neuroprotection and Neuroplasticity in Romania, Prof. Gelu Onose, MD, President Co-founder of the Romanian Society for Neurorehabilitation and Prof. Dorel Săndesc, MD, Secretary of State in the Ministry of Health-Care and President of the Romanian Society of Anesthesia and Intensive Care.

The Congress had great international participation, reuniting well-known specialists from Argentina, Hong Kong, Austria, Switzerland, the USA, Germany, and the Netherlands. Dafin Mureșanu – Romania, Wai Poon – Hong Kong, Volker Hömberg – Germany and Suhail Abdulla Al-Rukn – United Arab Emirates were in charge of the welcome address at this important event.

**Fig. 1:**
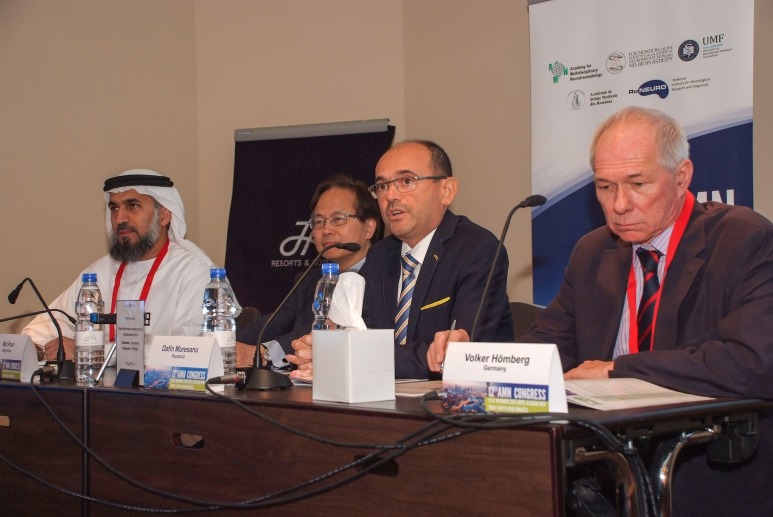
Welcome Address: from left to right Suhail Abdulla Al-Rukn – United Arab Emirates, Wai Poon – Hong Kong, Dafin Mureșanu – Romania, and Volker Hömberg – German

Prof. Dafin Mureșanu, MD, presented the advances in brain protection and recovery in traumatic brain injury.

**Fig. 2:**
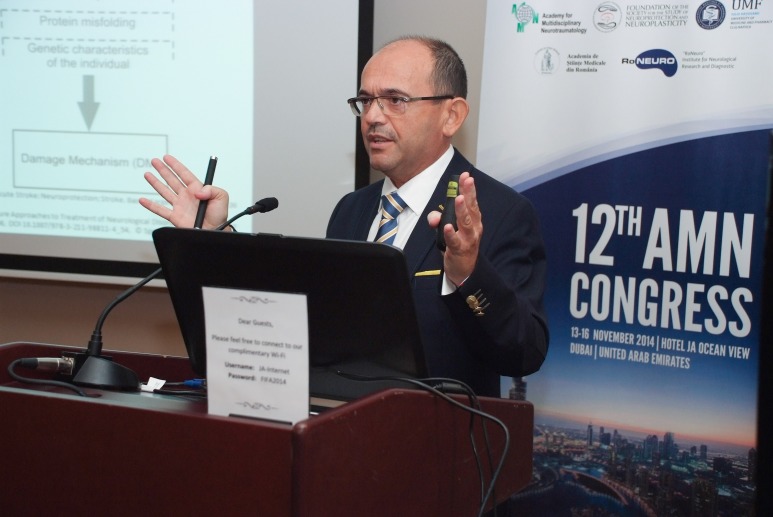
Prof. Dafin Mureșanu, MD, President of the SSNN and the Romanian Society of Neurology

**Fig. 3:**
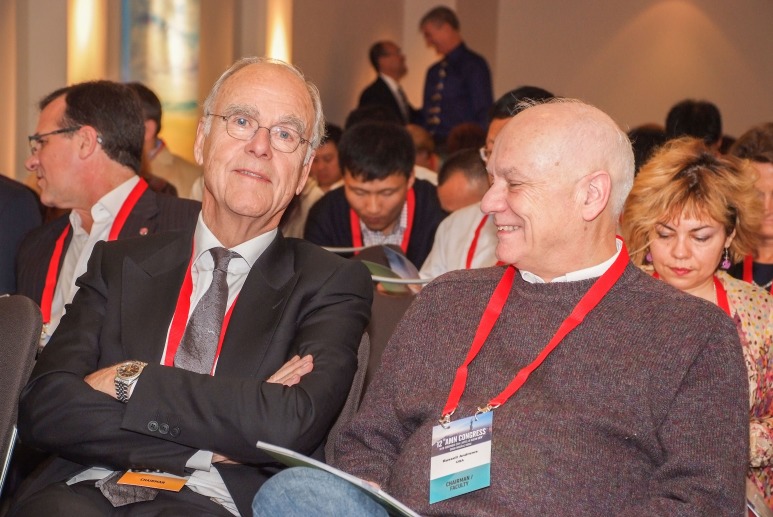
Klaus von Wild – Founding & Honorary President of the AMN and Russell Andrews – Ames Associate (Smart Systems & Nanotechnology), National Aeronautics and Space Administration (NASA) Ames Research Center, California, USA

The well-known specialist Volker Hömberg, Chairman of the scientific program committee of the AMN, from the Department of Neurology, Heinrich Heine University, Dusseldorf, and one of the representatives of Germany, discussed the future of scientific meetings: what new formats are needed.

**Fig. 4:**
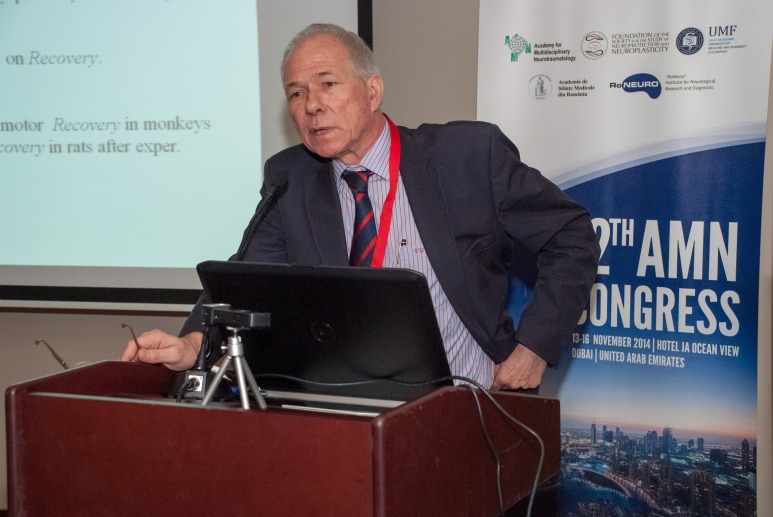
Volker Hömberg – Secretary General of World Federation for NeuroRehabilitation, Germany

Jan-Peter Jantzen, Head of the Department of Anesthesiology, Intensive Care Medicine and Pain Management, Academic Teaching Hospital Hannover, Nordstadt, Germany, presented, during the debates session, the problem of ICU management of traumatic brain injury - therapeutic coma, bringing important arguments for the application of this method.

**Fig. 5:**
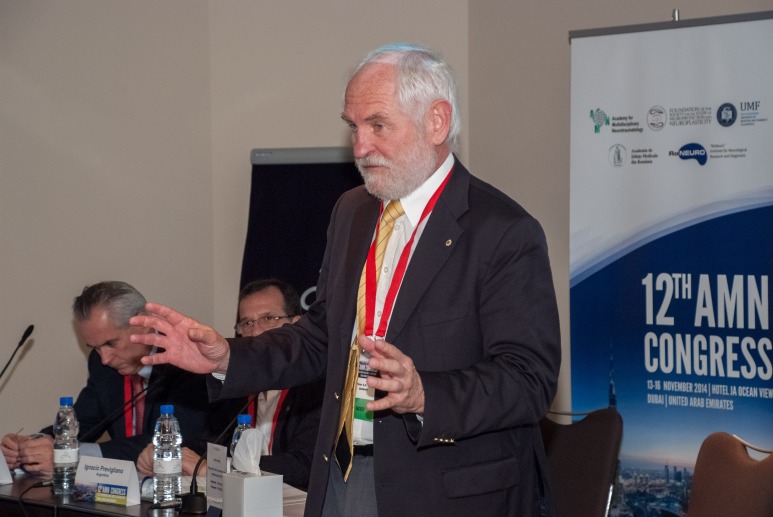
Jan-Peter Jantzen, Head of the Department of Anesthesiology, Intensive Care Medicine and Pain Management, Germany

Moreover, Johannes Vester, Department of Biometry and Clinical Research, IDV Data Analysis and Study Planning, Krailling, Germany, presented the multidimensional approach in clinical neuroscience research – advances and challenges.

**Fig. 6:**
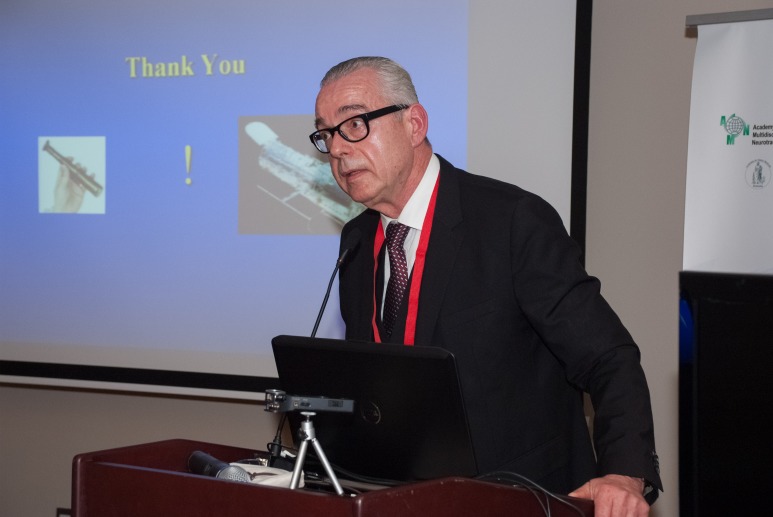
Johannes Vester, Department of Biometry and Clinical Research, IDV Data Analysis and Study Planning, Krailling, Germany

Dorel Săndesc, Secretary of State in the Ministry of Health-Care in Romania and President of the Romanian Society of Anesthesia and Intensive Care (Romania) underlined the most important aspects of the brain death concept as well as organ transplantation.

**Fig. 7:**
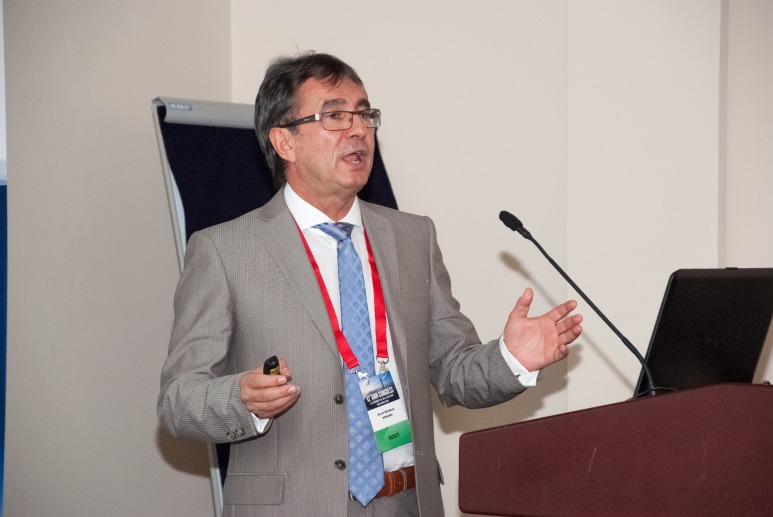
Dorel Săndesc, Secretary of State in the Ministry of Health-Care in Romania and President of the Romanian Society of Anesthesia and Intensive Care, Romania

Prof. Gelu Onose, MD, President and Co-founder of the Romanian Society for Neurorehabilitation in Romania discussed neuroprotection – with the use of some main related molecules – in patients with subacute/ subchronic conditions following severe central nervous system lesions.

**Fig. 8:**
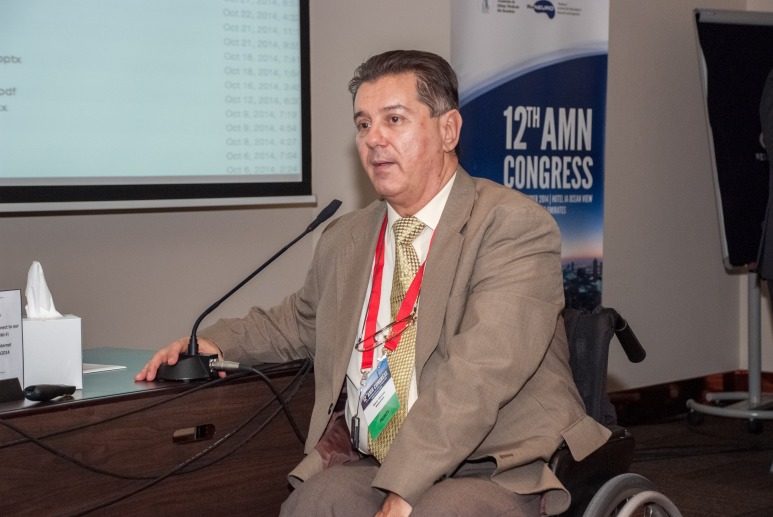
Prof. Gelu Onose, MD, President and Co-founder of the Romanian Society for Neurorehabilitation in Romania

Russell Andrews, Ames Associate, Ames Research Center in California, one of the representatives of the USA, discussed the disaster response: an opportunity to improve global healthcare in the 21st century, and Karin Diserens, Head of the Unit for acute Neurorehabilitation, Service de Neurologie, Departement Neurosciences Clinique, Lausanne, the representative of Switzerland, discussed the disorders of consciousness in early neurorehabilitation.

**Fig. 9:**
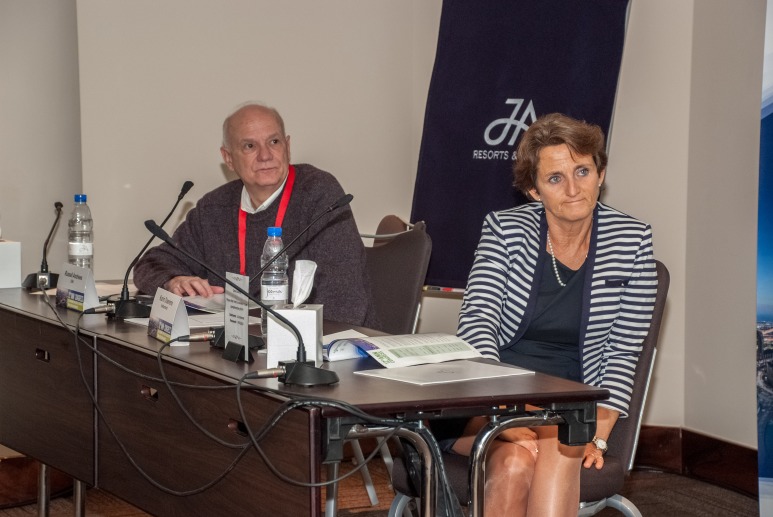
Russell Andrews, Ames Associate, Ames Research Center in California, NASA, SUA & Karin Diserens – Head of the Unit for acute Neurorehabilitation, Service de Neurologie, Departement Neurosciences Clinique, Lausanne, Switzerland

Nicole von Steinbüchel, Vice President of the AMN, Institute of Medical Psychology and Medical Sociology, University Medical Center, Georg-August University, Göttingen, Germany, discussed the outcome of individuals after mild traumatic brain injury.

**Fig. 10:**
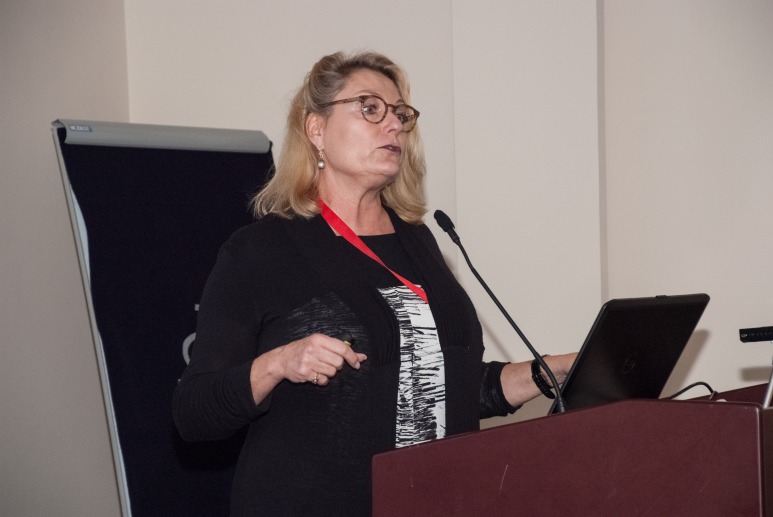
Nicole von Steinbüchel, Vice President of AMN, Institute of Medical Psychology and Medical Sociology, University Medical Center, Georg-August University, Göttingen, Germany

Thanks to an excellent organization and organizational team, which has outdone itself, the Congress had the expected outcome, ensuring exquisite accommodations and working conditions to all the participants. After the end of the highly interesting and complex scientific sessions, the participants visited the most important places in Dubai.

In addition, we should mention the contribution of the “RoNeuro” Institute for Neurological Research and Diagnostics of “Iuliu Hatieganu” University of Medicine and Pharmacy in Cluj-Napoca, Romania, as well as the contribution of the Academy of Medical Sciences in Romania in the process of organizing the resonant congress.

**Executive Editor** **Assoc. Prof. Dr. Eng. Victor Purcarea**

